# Dietary Supplementation with Multispecies Probiotic Fermented Broth Improves Nutrient Digestibility and Carcass Traits While Modulating the Cecal Microbiota and Thigh Muscle Fatty Acid Profile in Yellow-Feathered Broilers

**DOI:** 10.3390/ani16172768

**Published:** 2026-09-03

**Authors:** Haiqing Gan, Youping Tan, Dan Zhong, Sirui Li, Huange Xie, Xingguo Huang, Dun Deng, Qianglin Liu, Qiyu Tian, Lingyuan Yang

**Affiliations:** 1 College of Animal Science and Technology, Hunan Agricultural University, Changsha 410128, China; 2 Yuelushan Laboratory, Changsha 410128, China; 3 Tangrenshen Group Co., Zhuzhou 412007, China

**Keywords:** multispecies probiotic fermented broth, yellow-feathered broilers, apparent nutrient digestibility, carcass traits, cecal microbiota, thigh muscle fatty acid composition

## Abstract

This study evaluated the effects of dietary supplementation with 4% multispecies probiotic fermented broth (MPFB) on the apparent nutrient digestibility, carcass traits, serum immune indices, intestinal morphology, cecal microbiota, and thigh muscle nutrient composition in 815-strain yellow-feathered broilers. A total of 400 healthy 4-day-old broilers with similar body weight and a 1:1 sex ratio were randomly assigned to the control group with basal diet (CON) or the MPFB group with 4% MPFB. The results showed that MPFB significantly increased the apparent digestibility of crude protein and calcium (*p* < 0.05), and that of crude fiber and phosphorus (*p* < 0.01). The MPFB group had a higher thigh muscle yield and a lower abdominal fat yield (*p* < 0.05), while carcass yield tended to increase (*p* = 0.071). MPFB also elevated serum immunoglobulin M and interleukin-2 concentrations (*p* < 0.05), increased duodenal villus height (*p* < 0.05), reduced duodenal crypt depth (*p* < 0.05), and increased the villus height to crypt depth ratio in the duodenum, jejunum, and ileum (*p* < 0.05). Cecal microbiota analysis showed that MPFB significantly enriched *Ruminococcus gauvreauii*_group, *Ruminococcus torques*_group, and *Turicibacter* (*p* < 0.05), while *Bacteroides* tended to increase (*p* = 0.093), which were positively correlated with the improved nutrient digestibility and duodenal morphology. In thigh muscle, MPFB tended to increase cysteine content (*p* = 0.081), decreased C14:0 (*p* < 0.05), C16:0 and total saturated fatty acids (*p* < 0.01), increased C20:2 (*p* < 0.05), and tended to decrease C12:0 and C16:1 but increase C18:2n6c and total polyunsaturated fatty acid (*p* < 0.10). In conclusion, dietary supplementation with 4% MPFB improved nutrient utilization and selected carcass traits and modulated the thigh muscle fatty acid profile in yellow-feathered broilers, which are potentially associated with improved intestinal morphology and modulation of the cecal microbiota.

## Simple Summary

Multispecies probiotic fermented broth (MPFB) is a liquid fermentation product containing probiotics and fermentation-derived bioactive compounds. This study showed that dietary supplementation with 4% MPFB improved nutrient digestibility, carcass traits, and intestinal morphology, altered the cecal microbial composition, and modified the thigh muscle fatty acid profile in 815-strain yellow-feathered broilers. MPFB improved nutrient digestibility, selected carcass traits, immune status, and intestinal morphology, while modulating the cecal microbiota and thigh muscle fatty acid composition. These findings indicate that MPFB could serve as a functional fermented feed additive to enhance nutrient utilization, intestinal health, and the nutritional quality of meat in yellow-feathered broiler production.

## 1. Introduction

Restrictions on the use of antibiotic growth promoters in poultry production have increased interest in nutritional strategies that safely and effectively improve intestinal health. Probiotics are widely used in broiler diets because of their capacity to regulate the gut microbiota, enhance immune responses, and improve nutrient utilization [1]. Recent studies suggest that probiotic fermentation products, including probiotic fermented broths, may provide functional benefits beyond those of direct-fed probiotics or multispecies probiotic preparations [2,3]. These products provide viable microorganisms together with fermentation-derived bioactive compounds, including organic acids, small peptides, enzymes, and soluble nutrients, that may improve the intestinal microenvironment, nutrient digestibility, and gut function [4]. A previous study showed that maternal supplementation with a compound probiotic-fermented broth containing *Lactobacillus plantarum* and *Saccharomyces cerevisiae* can modulate immune responses, improve intestinal morphology, and reshape gut microbial composition in piglets [5]. Similarly, spraying *Lactobacillus plantarum* P-8 fermented broth enhanced the antioxidant capacity and immunoregulatory status and improved intestinal homeostasis in neonatal piglets by modulating the gut microbial structure [6]. A previous study in yellow-feathered broilers reported that dietary supplementation with 3% or 4% *Lactiplantibacillus plantarum* fermentation products improved growth performance and feed conversion, reduced gastrointestinal pH, and modulated the cecal microbiota [7]. However, information on the application of multispecies probiotic fermented broth (MPFB) in yellow-feathered broilers remains limited. Therefore, this study evaluated the effects of dietary supplementation with 4% MPFB on carcass traits, meat quality, apparent nutrient digestibility, serum immune indices, intestinal morphology, free amino acid and fatty acid composition in the thigh muscle and cecal microbiota in 815-strain yellow-feathered broilers.

## 2. Materials and Methods

### 2.1. Ethics Statement

All experimental procedures were approved by the Biomedical Research Ethics Committee of Hunan Agricultural University (No. 202620).

### 2.2. Preparation of MPFB

The compound probiotic preparation used to produce the MPFB was supplied as a powder and contained *Lactobacillus plantarum* at 3.5 × 10^5^ CFU/g, *Bacillus subtilis* at 1.5 × 10^6^ CFU/g, and *Saccharomyces cerevisiae* at 5.0 × 10^2^ CFU/g. During the experimental period, approximately 4 L of MPFB was prepared per batch. Briefly, 100 g of brown sugar, 100 g of whey powder, and 4 g of the compound probiotic preparation were added to 3.8 kg of water and mixed thoroughly. The mixture was then transferred to a sealed 6 L fermentation vessel and fermented at ambient temperature for 48 h. Staggered fermentation batches were prepared throughout the trial to ensure a daily supply of freshly prepared broth. Before feeding, MPFB was incorporated into the basal diet at 4% on an as-fed basis and mixed thoroughly to ensure uniform distribution.

### 2.3. Experimental Design and Diets

The feeding trial was conducted at Zhuzhou Qunfeng Farm in Zhuzhou, China. A total of 400 healthy 4-day-old yellow-feathered broilers of the 815 strain with similar body weight (BW) and an equal sex ratio were randomly assigned to two treatment groups. The CON group was fed a basal diet, whereas the MPFB group was fed the basal diet supplemented with 4% MPFB. Each group had 10 replicates, with 20 chickens per replicate. The basal diets were formulated according to the nutrient requirements specified by the NRC (1994) [8], and their ingredient composition and nutrient levels are shown in Table 1. The trial was conducted in three phases: 9 to 21 days, 22 to 42 days, and 43 to 56 days. During the early phase, the chickens were fed with crushed pellets, whereas during the later phases, they were fed mash diets. The chickens were raised on the floor pens with free access to feed and water. The housing temperature was maintained at 25 to 30 °C. Natural ventilation was ensured, and the chickens received 16 h of light daily. All other management practices and vaccination procedures followed the standard protocols of the farm.

### 2.4. Carcass Traits

At 56 days of age, broilers were fasted for 12 h before slaughter. Ten broilers (one from each replicate) were randomly selected. After exsanguination and defeathering, the carcass yield, eviscerated carcass yield, semi-eviscerated carcass yield, breast muscle yield, abdominal fat yield, and thigh muscle yield were determined, as described previously [9].

### 2.5. Meat Quality

At 56 days of age, the left breast and thigh muscles were collected for meat quality analysis. The meat color, pH, drip loss, cooking loss, shear force, and water loss rate were measured, as described previously [10].

### 2.6. Determination of Amino Acids in Thigh Muscles

A 2.5 g freeze-dried thigh muscle sample was homogenized in 25 mL of 0.01 N hydrochloric acid, followed by ultrasonic extraction for 30 min. The homogenate was then filtered, and 2 mL of the filtrate was mixed with 2 mL of 8% sulfosalicylic acid. After standing for 15 min, the mixture was centrifuged at 10,000× *g* for 10 min. The supernatant was then filtered through a 0.22 μm filter. Amino acid analysis was performed using a Hitachi LA8080 ultra-high-speed automatic amino acid analyzer (Hitachi High-Tech Science Corporation, Tokyo, Japan).

### 2.7. Determination of Fatty Acid Composition in Thigh Muscles

A 0.5 g freeze-dried thigh muscle sample was extracted for 24 h with 4 mL of a benzene–petroleum ether mixture (1:1, *v*/*v*). The resulting extract was then esterified with 4 mL of a 0.4 mol/L potassium hydroxide–methanol solution. After shaking for 3 min and standing 30 min, ultrapure water was added to induce phase separation. The upper phase was collected, and anhydrous sodium sulfate was added. Next, 200 μL of the sample was diluted with 800 μL of hexane and filtered through a 0.22 μm filter membrane. The sample was then analyzed using a Thermo Scientific™ Q Exactive™ Plus hybrid quadrupole-Orbitrap™ mass spectrometer system (Thermo Fisher Scientific, Waltham, MA, USA).

### 2.8. Nutrient Digestibility

Feed samples were collected weekly, and fecal samples from each replicate pen were collected at 53, 54, and 55 days of age. The hydrochloric acid–insoluble ash, crude ash, crude protein (CP), crude fiber (CF), ether extract (EE), calcium (Ca), and phosphorus (P) contents of the feed and fecal samples were determined according to the corresponding Chinese National Standards (GB/T 23742-2009, GB/T 6438-2007, GB/T 6432-2018, GB/T 6434-2006, GB/T 6433-2006, GB/T 6436-2018, and GB/T 6437-2018 [11,12,13,14,15,16,17]), respectively. Gross energy (GE) was measured using the WZR-1T-B automatic calorimeter (Changsha Bente Instrument Co., Ltd., Changsha, China).

### 2.9. Blood Immune Parameters

Serum concentrations of immunoglobulin A (IgA), immunoglobulin G (IgG), immunoglobulin M (IgM), and interleukin-2 (IL-2) were determined using enzyme-linked immunosorbent assay kits (Jiangsu Enzyme Immuno Industry Co., Ltd., Yancheng, China) according to the manufacturer’s instructions.

### 2.10. Intestinal Morphology

At 56 days of age, duodenal, jejunal, and ileal segments were collected and fixed in 4% paraformaldehyde for 24 h. The fixed tissues were embedded in paraffin, sectioned, and stained with hematoxylin and eosin. The intestinal mucosal morphology was then examined under a light microscope. The villus height (VH) and crypt depth (CD) were measured using Image-Pro Plus 6.0 software (Media Cybernetics, Inc., Rockville, MD, USA). For each section, 10 well-oriented intact villi and their corresponding crypts were measured, and the villus height-to-crypt depth ratio (VH/CD) was calculated.

### 2.11. Cecal Microbiota Analysis

Six cecal digesta samples per treatment were selected from each group for 16S rRNA gene sequencing. Total microbial DNA was extracted using the TransGen AP221-02 kit (TransGen Biotech Co., Ltd., Beijing, China). The V3–V4 region of the bacterial 16S rRNA gene was amplified using the primers 338F (5′-ACTCCTACGGGAGGCAGCAG-3′) and 806R (5′-GGACTACHVGGGTWTCTAAT-3′). Amplicon sequencing was performed on the Illumina MiSeq platform (Illumina, Inc., San Diego, CA, USA) by Majorbio Bio-Pharm Technology Co., Ltd. (Shanghai, China). Paired-end reads were merged and quality-filtered to remove low-quality, ambiguous, and chimeric sequences. High-quality sequences were clustered into operational taxonomic units (OTUs) at 97% sequence similarity. Good’s coverage was calculated to assess the sequencing completeness. Alpha diversity, taxonomic composition, principal coordinate analysis, and OTU-based analyses were performed using QIIME 1.9.1 and R 4.5.3. No additional low-abundance filtering was applied before differential abundance analysis. Differences between the CON and MPFB groups were evaluated using the Wilcoxon rank-sum test, with *p* < 0.05 considered significant. Linear discriminant analysis effect size (LEfSe) was performed using an LDA score threshold of 2.0 and *p* < 0.05.

### 2.12. Statistical Analysis

Data were analyzed using an independent-samples t-test in SPSS 25.0 (IBM SPSS Statistics, Armonk, NY, USA). The results are presented as means ± SEM. *p* < 0.05 was considered statistically significant, whereas 0.05 ≤ *p* < 0.10 was considered a trend.

## 3. Results

### 3.1. Growth Performance

As shown in Table 2, dietary supplementation with MPFB significantly increased the average daily feed intake (ADFI) during d 43 to 56 (*p* < 0.05).

### 3.2. Carcass Traits

As shown in Table 3, broilers in the MPFB group had a higher thigh muscle yield and a lower abdominal fat yield than those in the CON group (*p* < 0.05), while the carcass yield tended to be higher (*p* = 0.071).

### 3.3. Meat Quality

As shown in Table 4, the MPFB group tended to have a lower thigh muscle b* value than the CON group (*p* = 0.073).

### 3.4. Free Amino Acid Contents in the Thigh Muscle

As shown in Table 5, the Cys content of the thigh muscles tended to be higher in the MPFB group than in the CON group (*p* = 0.081).

### 3.5. Fatty Acid Composition in the Thigh Muscle

As shown in Table 6, compared with the CON group, MPFB markedly decreased C16:0 and total SFA (*p* < 0.01), decreased C14:0 (*p* < 0.05), and increased C20:2 (*p* < 0.05). Moreover, MPFB tended to decrease C12:0 (*p* = 0.097) and C16:1 (*p* = 0.064) and tended to increase C18:2n6c (*p* = 0.057) and total PUFA (*p* = 0.054).

### 3.6. Apparent Nutrient Digestibility

As presented in Table 7, MPFB increased the apparent digestibility of CP and Ca (*p* < 0.05) and that of CF and P (*p* < 0.01).

### 3.7. Serum Immune Indices

As shown in Table 8, the serum IgM and IL-2 concentrations were significantly higher in the MPFB group than in the CON group (*p* < 0.05), whereas IgA showed an increasing tendency (*p* = 0.056).

### 3.8. Intestinal Morphology

Table 9 shows that MPFB increased the duodenal VH and the duodenal VH/CD ratio and reduced the duodenal CD (*p* < 0.05). In addition, the VH/CD ratios in the jejunum and ileum were also significantly higher (*p* < 0.05), with a tendency toward higher VH in the jejunum (*p* = 0.073) and ileum (*p* = 0.059).

### 3.9. Cecal Microbiota

**Figure 1:**
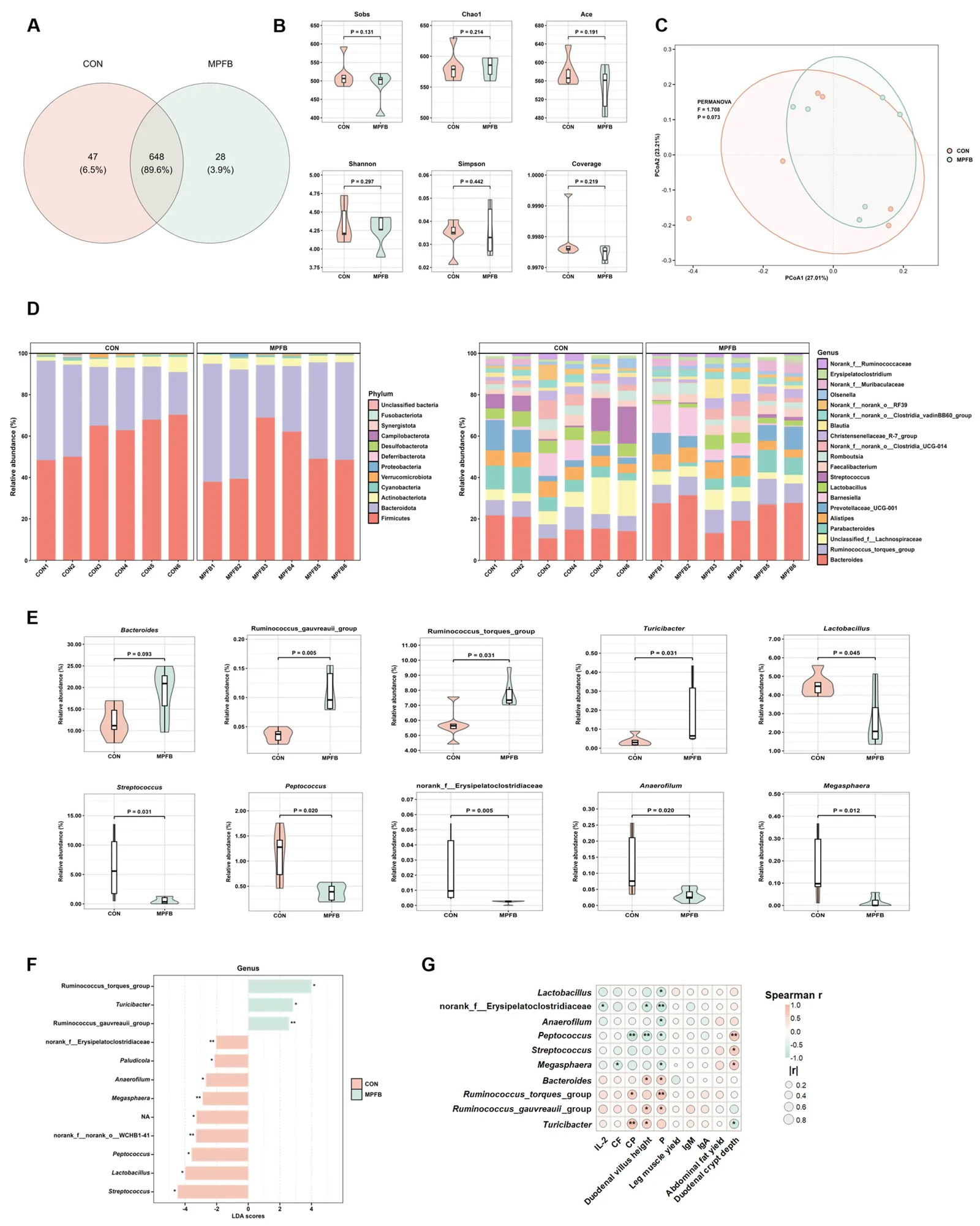
Effects of dietary supplementation with MPFB on the cecal microbiota of yellow-feathered broilers (*n* = 6 per group). (**A**) Venn diagram at the OTU level. (**B**) Alpha-diversity indices of the cecal microbiota. (**C**) Principal coordinate analysis (PCoA) of microbial community structure. (**D**) Relative abundance of cecal microbiota at the phylum and genus levels. (**E**) Differentially abundant genera between the CON and MPFB groups. (**F**) LEfSe analysis of the major bacterial taxa enriched in each group (LDA score ≥ 2.0 and *p* < 0.05), * indicates *p* < 0.05, and ** indicates *p* < 0.01. (**G**) Spearman correlation analysis between differential bacterial genera and apparent nutrient digestibility or intestinal morphology indices. CON = basal diet; MPFB = basal diet supplemented with 4% multispecies probiotic fermented broth; OTU = operational taxonomic unit; PCoA = principal coordinate analysis; LDA = linear discriminant analysis; LEfSe = linear discriminant analysis effect size. In panel (**G**), * indicates *p* < 0.05, and ** indicates *p* < 0.01.

As shown in Figure 1A, 648 OTUs were shared between the CON and MPFB groups, accounting for 89.6% of the total OTUs. No significant differences were observed in the alpha diversity indices between the two groups (Figure 1B; *p* > 0.05). Principal coordinate analysis (PCoA) revealed a tendency toward separation in microbial community structure (Figure 1C; PERMANOVA, *p* = 0.077). At the phylum level, the microbiota of both groups was dominated by Firmicutes and Bacteroidota (Figure 1D). At the genus level, the relative abundances of *Bacteroides*, *Ruminococcus gauvreauii*_group, *Ruminococcus torques*_group, and *Turicibacter* were significantly increased in the MPFB group, whereas those of *Lactobacillus*, *Streptococcus*, *Peptococcus*, norank_f__Erysipelatoclostridiaceae, *Anaerofilum*, and *Megasphaera* were significantly decreased compared with the CON group (Figure 1D,E; *p* < 0.05). LEfSe analysis further identified *Ruminococcus torques*_group, Turicibacter, and *Ruminococcus gauvreauii*_group as biomarkers of the MPFB group (Figure 1F). Correlation analysis further showed that Bacteroides was positively correlated with the duodenal villus height and apparent phosphorus digestibility, *Ruminococcus torques*_group was positively correlated with the apparent crude protein and phosphorus digestibility, and *Ruminococcus gauvreauii*_group was positively correlated with the duodenal villus height and apparent phosphorus digestibility (Figure 1G; *p* < 0.05). In addition, Turicibacter was positively correlated with apparent crude protein digestibility and duodenal villus height but negatively correlated with duodenal crypt depth (*p* < 0.05). In contrast, norank_f__Erysipelatoclostridiaceae, Peptococcus, and Megasphaera showed significant correlations in the opposite direction with some nutrient digestibility and intestinal morphology indices, whereas Streptococcus was positively correlated with the duodenal crypt depth (*p* < 0.05).

## 4. Discussion

In the present study, MPFB was produced by the co-fermentation of *Bacillus subtilis*, *Lactobacillus plantarum*, and *Saccharomyces cerevisiae*. Its beneficial effects may be attributed to the combined actions of viable microorganisms and fermentation-derived bioactive compounds, such as organic acids, enzymes, peptides, and other metabolites [18]. These probiotic species and related fermented products have been reported to enhance immune function, modulate gut microbiota, and improve nutrient digestion and absorption in poultry [19,20]. Consistently, MPFB supplementation significantly increased the ADFI in yellow-feathered broilers from 43 to 56 d of age, which may be partly associated with improved diet palatability and gastrointestinal conditions in the later growth phase [21].

Dietary MPFB supplementation altered carcass traits in yellow-feathered broilers, as reflected by increased thigh muscle yield and reduced abdominal fat yield, while the carcass yield tended to increase. The increased thigh muscle yield may be associated with enhanced nutrient utilization, as indicated by the improved apparent digestibility of crude protein, calcium, crude fiber, and phosphorus in the MPFB group. Meanwhile, the reduction in abdominal fat yield suggests decreased lipid deposition, because abdominal fat is commonly used as an indicator of lipid deposition in broilers [22,23]. Previous studies have reported that *Bacillus subtilis* supplementation reduces abdominal fat deposition, whereas *Lactiplantibacillus plantarum*-fermented feed decreases abdominal fat weight by downregulating lipogenesis-related genes and upregulating lipolysis-related genes [24,25]. Collectively, these findings suggest that MPFB may improve the carcass composition by promoting nutrient utilization and muscle deposition while reducing lipid accumulation.

MPFB supplementation also tended to reduce the b* value of the thigh muscle at 45 min postmortem, indicating a possible decrease in yellowness. Previous studies have shown that probiotic supplementation, including *Bacillus subtilis*, can affect meat color parameters in broilers [26,27]. However, the mechanism by which MPFB improves chicken meat color remains unclear.

Dietary MPFB supplementation also modified the amino acid and fatty acid composition of thigh muscle. In the present study, MPFB tended to increase the Cys content, decreased C14:0, C16:0, and total SFA, increased C20:2, and tended to decrease C12:0 and C16:1, while increasing C18:2n6c and total PUFA. Cys is an important sulfur-containing amino acid involved in glutathione synthesis and muscle redox metabolism [28]. The reduction in C12:0, C14:0, and C16:0 may be nutritionally relevant, because these saturated fatty acids are closely associated with the atherogenic potential of meat lipids [29], whereas the PUFA/SFA ratio is commonly used as an indicator of lipid nutritional quality [30]. Moreover, C18:2n-6 is one of the predominant n-6 PUFA in poultry meat, and C20:2 can be considered a potential elongation-related product of C18:2n-6. Thus, the significant increase in C20:2, together with the tendency toward higher C18:2n6c, may reflect changes in n-6 PUFA deposition or elongation-related metabolism [31,32]. In contrast, Yang et al. reported that a fermented product prepared with Aspergillus niger, *Saccharomyces cerevisiae*, and *Bacillus subtilis* did not significantly affect the overall fatty acid composition of broiler breast muscle [33]. Such discrepancies may be attributable to differences in microbial composition, fermentation conditions, product characteristics, bird age, and feeding duration among studies. Collectively, the present results suggest that MPFB may improve the lipid nutritional value of thigh muscle; however, the mechanisms underlying these changes remain to be elucidated.

The improvements in carcass traits and muscle nutrient composition may be associated with enhanced nutrient utilization. In the present study, MPFB increased the apparent digestibility of CP, Ca, CF and P, suggesting the improved utilization of protein, minerals, and fibrous components. Similarly, Ning et al. [3] reported that *Lactobacillus plantarum* fermented broth significantly increased the apparent nutrient digestibility and improved the intestinal morphology in Xiangdong chickens. These effects may be attributed to coordinated actions of organic acids and other fermentation-derived bioactive compounds, which may improve the gastrointestinal environment and facilitate the degradation and utilization of complex nutrients [32,34,35].

The effects of MPFB on serum immune indices and intestinal morphology further support its role in improving intestinal function. In the present study, MPFB significantly increased the serum IgM and IL-2 concentrations and tended to increase IgA, suggesting that it may modulate humoral, mucosal, and cellular immunity. This is consistent with previous findings that maternal supplementation with a compound probiotic fermented broth containing *Lactobacillus plantarum* and *Saccharomyces cerevisiae* significantly increased plasma IgA, as well as the jejunal IL-10, IFN-α, and sIgA and ileal sIgA levels [5]. Meanwhile, MPFB significantly increased the duodenal villus height, reduced the duodenal crypt depth, and increased the VH/CD ratio in the duodenum, jejunum, and ileum, indicating that MPFB improved the intestinal morphology. Increased villus height and reduced crypt depth are generally considered indicative of improved intestinal mucosal status and enhanced absorptive capacity [36]. In addition, postbiotics, which are bioactive metabolites produced during probiotic growth, have also been shown to improve small intestinal morphology in yellow-feathered broilers [37,38]. Therefore, MPFB may support nutrient digestion and absorption through improvements in intestinal morphology and modulation of selected serum immune-related indices.

The gut microbiota plays an important role in nutrient metabolism, intestinal development, and the maintenance of host homeostasis [39]. In the present study, MPFB had no significant effect on the α-diversity or β-diversity of the cecal microbiota in broilers. In both groups, Firmicutes and Bacteroidota were the dominant phyla, which is consistent with the general compositional characteristics of the broiler cecal microbiota [40]. Notably, *Bacteroides*, *Ruminococcus gauvreauii*_group, *Ruminococcus torques*_group, and *Turicibacter* were significantly enriched in the MPFB group. Previous studies have shown that *Bacteroides* has a strong capacity to degrade complex carbohydrates [41], Ruminococcus-related taxa are involved in substrate fermentation and metabolite production [42], and *Turicibacter* is closely associated with host intestinal metabolic regulation [43]. Correlation analysis further revealed that these enriched bacterial groups were positively correlated with the apparent digestibility of crude protein and phosphorus, as well as the duodenal villus height, while *Turicibacter* showed a significant negative correlation with the duodenal crypt depth. These findings suggest that the enrichment of taxa associated with digestive and metabolic functions may partly explain the improved nutrient utilization efficiency and intestinal morphology observed in the MPFB group. In contrast, *Lactobacillus*, *Streptococcus*, *Peptococcus*, norank_f__Erysipelatoclostridiaceae, *Anaerofilum*, and *Megasphaera* were significantly reduced in the MPFB group, and some of these genera were negatively correlated with apparent nutrient digestibility and intestinal morphological indices. Their decreased abundance may be related to competitive exclusion following the introduction of exogenous probiotics [44]. Notably, *Lactobacillus* predominantly colonizes the foregut in chickens, and its lower abundance in the cecum may also reflect microbial adaptation to segment-specific intestinal environments [45]. In addition, the decline in *Streptococcus*, which includes some opportunistic pathogenic species [46], may partly reflect an improvement in the intestinal microecological environment. Collectively, MPFB supplementation improved nutrient digestibility and was accompanied by favorable changes in intestinal morphology and cecal microbiota composition. However, as only one MPFB inclusion level was evaluated, its dose–response relationship and optimal inclusion level remain to be determined.

## 5. Conclusions

In conclusion, under the conditions of the present study, dietary supplementation with 4% MPFB improved nutrient utilization and carcass traits and favorably modified the thigh muscle fatty acid profile in yellow-feathered broilers. These changes were accompanied by enhanced intestinal morphology and alterations in the cecal microbiota.

## Figures and Tables

**Table 1:** Ingredients’ composition and nutrient levels of basal diets (as-fed basis).

Items	Age (Days)		
	Starter	Grower	Finisher
	9 to 21 Days	22 to 42 Days	43 to 56 Days
**Ingredients (%)**			
Corn	46.30	39.00	39.60
Wheat	5.00	10.00	10.00
Barley	6.00	11.00	10.00
Soybean meal	24.60	19.00	16.00
Extruded full-fat soybean	2.50	4.00	5.00
Corn gluten meal	4.50	4.50	5.41
Cottonseed meal	3.00	3.00	3.00
Calcium hydrogen phosphate	1.60	1.05	0.90
Limestone	0.82	1.07	1.09
Soybean oil	2.68	4.38	6.00
Premix ^1^	3.00	3.00	3.00
Total	100	100	100
**Nutrient levels ^2^**			
ME, kcal/kg	2855	2960	3089
CP, %	17.01	16.13	15.85
EE, %	2.71	3.65	4.56
CF, %	1.71	1.66	1.58
Available P, %	0.58	0.44	0.40
Ca, %	1.02	0.93	0.87
Lys, %	0.62	0.60	0.54
Met, %	0.55	0.49	0.45

ME = metabolizable energy; CP = crude protein; EE = ether extract; CF = crude fiber; Available P = available phosphorus; Ca = calcium; Lys = lysine; Met = methionine. ^1^ Premix provided the following per kilogram of diet: VA 6000 IU, VD3 2500 IU, VE 25 mg, VK3 2.25 mg, VB1 1.8 mg, VB2 7 mg, VB6 4 mg, VB12 0.2 mg, D-pantothenic acid 12 mg, nicotinic acid 35 mg, biotin 0.14 mg, folic acid 0.8 mg, Cu 11 mg, Zn 70 mg, Fe 60 mg, Mn 115 mg, Se 0.30 mg, and I 0.4 mg. ^2^ Nutrient levels were calculated.

**Table 2:** Effects of MPFB on growth performance in yellow-feathered broilers.

Items	Day	CON	MPFB	SEM	*p*-Value
BW, g	9	124.83	125.28	1.40	0.877
	21	407.75	406.25	2.45	0.769
	42	1163.83	1119.95	13.27	0.449
	56	1748.45	1771.54	13.19	0.396
ADFI, g/d	9 to 21	40.81	40.85	0.20	0.925
	22 to 42	80.21	82.54	0.89	0.199
	43 to 56	125.27	130.53	1.24	0.028
	9 to 56	81.46	83.30	0.60	0.130
ADG, g/d	9 to 21	21.76	21.61	0.14	0.599
	22 to 42	33.48	33.99	0.40	0.542
	43 to 56	44.97	50.12	1.94	0.191
	9 to 56	34.55	35.03	0.27	0.396
FCR, g/g	9 to 21	1.88	1.89	0.01	0.349
	22 to 42	2.40	2.40	0.04	0.626
	43 to 56	2.56	2.64	0.02	0.118
	9 to 56	2.36	2.42	0.02	0.171

CON = basal diet; MPFB = basal diet supplemented with 4% multispecies probiotic fermented broth; ADFI = average daily feed intake; ADG = average daily gain; FCR = feed conversion ratio; SEM = standard error of the mean. Values are presented as means, *n* = 10 replicates/treatment.

**Table 3:** Effects of MPFB on carcass traits of yellow-feathered broilers (%).

Items	CON	MPFB	SEM	*p*-Value
Carcass yield	91.38	92.18	0.22	0.071
Eviscerated carcass yield	85.61	86.26	0.28	0.253
Semi-eviscerated carcass yield	70.22	70.67	0.33	0.505
Thigh muscle yield	17.32	21.86	0.94	0.011
Breast muscle yield	13.36	14.83	0.45	0.104
Abdominal fat yield	3.29	2.64	0.12	0.002

CON = basal diet; MPFB = basal diet supplemented with 4% multispecies probiotic fermented broth; SEM = standard error of the mean. Values are presented as means, *n* = 10 birds/treatment.

**Table 4:** Effects of MPFB on meat quality of yellow-feathered broilers.

Items	CON	MPFB	SEM	*p*-Value
Thigh muscle				
pH_24 h_	5.89	5.90	0.04	0.939
Water loss rate, %	30.26	28.06	0.95	0.259
Cooking loss, %	23.96	26.73	1.51	0.376
Shear force, N	13.45	11.24	0.68	0.106
Drip loss, %	9.01	9.83	0.53	0.462
L* (45 min)	12.11	11.55	0.36	0.454
a* (45 min)	−0.40	−0.45	0.03	0.490
b* (45 min)	0.48	0.38	0.03	0.073
Breast muscle				
pH_24 h_	5.67	5.76	0.04	0.283
Water loss rate, %	33.18	35.89	1.07	0.216
Cooking loss, %	18.30	18.62	0.54	0.781
Shear force, N	19.54	16.01	1.32	0.191
Drip loss, %	7.90	6.95	0.47	0.328
L* (45 min)	11.94	11.39	0.25	0.282
a* (45 min)	−0.35	−0.38	0.01	0.143
b* (45 min)	0.41	0.41	0.01	0.769

CON = basal diet; MPFB = basal diet supplemented with 4% multispecies probiotic fermented broth; L* = lightness; a* = redness; b* = yellowness; SEM = standard error of the mean. Values are presented as means, *n* = 10 birds/treatment.

**Table 5:** Effects of MPFB on free amino acid contents in the thigh muscle of yellow-feathered broilers (μg/g).

Items	CON	MPFB	SEM	*p*-Value
His	89.72	93.99	5.31	0.733
Thr	190.10	202.64	5.77	0.328
Lys	127.4	133.63	10.40	0.803
Met	39.97	38.26	0.72	0.275
Val	125.82	115.55	5.04	0.364
Ile	55.13	45.29	3.51	0.184
Leu	121.47	110.63	7.06	0.505
Phe	84.66	81.24	1.24	0.194
Ala	1191.56	1342.25	93.79	0.484
Pro	141.85	158.07	16.19	0.669
Tyr	72.44	71.12	2.29	0.808
Ser	221.67	210.40	7.26	0.500
Arg	221.05	214.68	9.47	0.776
Gly	373.62	400.27	19.67	0.559
Asp	27.53	25.06	7.30	0.887
Glu	321.92	245.46	41.47	0.417
Cys	7.21	13.69	1.91	0.081
Trp	25.60	25.27	1.12	0.899
TAA	3438.84	3527.50	151.98	0.805
EAA	834.37	821.25	26.58	0.835
NEAA	2147.92	2667.30	226.44	0.298
FAA	2350.60	2452.62	135.30	0.749

CON = basal diet; MPFB = basal diet supplemented with 4% multispecies probiotic fermented broth; His = histidine; Thr = threonine; Lys = lysine; Met = methionine; Val = valine; Ile = isoleucine; Leu = leucine; Phe = phenylalanine; Ala = alanine; Pro = proline; Tyr = tyrosine; Ser = serine; Arg = arginine; Gly = glycine; Asp = aspartic acid; Glu = glutamic acid; Cys = cysteine; Trp = tryptophan; TAA = total amino acids; EAA = essential amino acids; NEAA = nonessential amino acids; FAA = flavor amino acids; SEM = standard error of the mean. Values are presented as means, *n* = 3 birds/treatment.

**Table 6:** Effects of MPFB on fatty acid composition in the thigh muscle of yellow-feathered broilers (%).

Items	CON	MPFB	SEM	*p*-Value
C8:0	0.12	0.13	0.01	0.683
C10:0	0.09	0.09	0.00	0.207
C12:0	0.08	0.08	0.00	0.097
C14:0	1.40	1.24	0.04	0.030
C16:0	25.59	24.18	0.25	0.007
C17:0	0.15	0.15	0.01	0.750
C18:0	14.05	14.11	0.39	0.912
C20:0	0.20	0.22	0.01	0.471
SFA	42.38	40.19	0.35	0.005
C16:1	2.58	2.19	0.11	0.064
C17:1	0.11	0.11	0.01	0.604
C18:1n9t	0.13	0.13	0.01	0.845
C18:1n9c	41.72	41.51	0.69	0.834
C20:1	0.87	0.85	0.05	0.865
MUFA	44.66	44.75	0.66	0.923
C18:2n6c	10.81	12.57	0.53	0.057
C18:3n3	0.52	0.57	0.03	0.233
C20:2	0.50	0.58	0.02	0.033
C20:3n6	0.15	0.15	0.01	1.000
C20:4n6	0.96	1.17	0.11	0.258
PUFA	12.97	15.05	0.62	0.054

CON = basal diet; MPFB = basal diet supplemented with 4% multispecies probiotic fermented broth; SFA = saturated fatty acids; MUFA = monounsaturated fatty acids; PUFA = polyunsaturated fatty acids; SEM = standard error of the mean. Values are presented as means, *n* = 4 birds/treatment.

**Table 7:** Effects of MPFB on apparent nutrient digestibility of yellow-feathered broilers (%).

Items	CON	MPFB	SEM	*p*-Value
CP	71.19	73.40	0.45	0.010
EE	47.23	49.68	1.40	0.401
Ash	42.43	39.85	1.51	0.411
Ca	59.91	64.81	1.13	0.025
P	41.88	46.77	0.84	0.001
CF	24.85	31.72	1.18	0.001

CON = basal diet; MPFB = basal diet supplemented with 4% multispecies probiotic fermented broth; CP = crude protein; EE = ether extract; Ash = crude ash; Ca = calcium; P = phosphorus; CF = crude fiber; SEM = standard error of the mean. Values are presented as means, *n* = 10 replicates/treatment.

**Table 8:** Effects of MPFB on serum immune indices of yellow-feathered broilers.

Items	CON	MPFB	SEM	*p*-Value
IgA, μg/mL	165.57	202.88	9.87	0.056
IgG, μg/mL	1319.65	1557.72	91.97	0.204
IgM, μg/mL	548.93	699.15	31.65	0.013
IL-2, pg/mL	120.45	161.27	9.72	0.031

CON = basal diet; MPFB = basal diet supplemented with 4% multispecies probiotic fermented broth; IgA = immunoglobulin A; IgG = immunoglobulin G; IgM = immunoglobulin M; IL-2 = interleukin-2; SEM = standard error of the mean. Values are presented as means, *n* = 10 birds/treatment.

**Table 9:** Effects of MPFB on the intestinal morphology of yellow-feathered broilers.

Items	CON	MPFB	SEM	*p*-Value
Duodenum				
VH, μm	814.35	963.52	28.03	0.004
CD, μm	81.43	66.84	2.57	0.002
VH/CD	10.08	14.48	0.69	<0.001
Jejunum				
VH, μm	816.89	914.53	27.35	0.073
CD, μm	83.06	76.38	2.28	0.147
VH/CD	9.92	12.11	0.45	0.010
Ileum				
VH, μm	826.96	919.36	24.73	0.059
CD, μm	77.17	70.89	2.26	0.170
VH/CD	10.46	12.58	0.39	0.003

CON = basal diet; MPFB = basal diet supplemented with 4% multispecies probiotic fermented broth; VH = villus height; CD = crypt depth; VH/CD = villus height-to-crypt depth ratio; SEM = standard error of the mean. Values are presented as means, *n* = 10 birds/treatment.

## Data Availability

The data presented in this study are available upon request from the corresponding author.
